# Tracing of Two *Pseudomonas* Strains in the Root and Rhizoplane of Maize, as Related to Their Plant Growth-Promoting Effect in Contrasting Soils

**DOI:** 10.3389/fmicb.2016.02150

**Published:** 2017-01-10

**Authors:** Carla Mosimann, Thomas Oberhänsli, Dominik Ziegler, Dinah Nassal, Ellen Kandeler, Thomas Boller, Paul Mäder, Cécile Thonar

**Affiliations:** ^1^Department of Environmental Sciences, Botany, Zürich-Basel Plant Science Center, University of BaselBasel, Switzerland; ^2^Research Institute of Organic Agriculture (FIBL)Frick, Switzerland; ^3^Mabritec AGRiehen, Switzerland; ^4^Institute of Soil Science and Land Evaluation, University of HohenheimStuttgart, Germany

**Keywords:** inoculation, MALDI-TOF, persistence, PGPR, *Pseudomonas* sp, qPCR, *Zea mays*

## Abstract

TaqMan-based quantitative PCR (qPCR) assays were developed to study the persistence of two well-characterized strains of plant growth-promoting rhizobacteria (PGPR), *Pseudomonas fluorescens* Pf153 and *Pseudomonas* sp. DSMZ 13134, in the root and rhizoplane of inoculated maize plants. This was performed in pot experiments with three contrasting field soils (Buus, Le Caron and DOK-M). Potential cross-reactivity of the qPCR assays was assessed with indigenous *Pseudomonas* and related bacterial species, which had been isolated from the rhizoplane of maize roots grown in the three soils and then characterized by Matrix-Assisted Laser Desorption Ionization (MALDI) Time-of-Flight (TOF) mass spectrometry (MS). Sensitivity of the qPCR expressed as detection limit of bacterial cells spiked into a rhizoplane matrix was 1.4 × 10^2^ CFU and 1.3 × 10^4^ CFU per gram root fresh weight for strain Pf153 and DSMZ 13134, respectively. Four weeks after planting and inoculation, both strains could readily be detected in root and rhizoplane, whereas only Pf153 could be detected after 8 weeks. The colonization rate of maize roots by strain Pf153 was significantly influenced by the soil type, with a higher colonization rate in the well fertile and organic soil of Buus. Inoculation with strain DSMZ 13134, which colonized roots and rhizoplane to the same degree, independently of the soil type, increased yield of maize, in terms of biomass accumulation, only in the acidic soil of Le Caron, whereas inoculation with strain Pf153 reduced yield in the soil Buus, despite of its high colonization rate and persistence. These results indicate that the colonization rate and persistence of inoculated *Pseudomonas* strains can be quantitatively assessed by the TaqMan-based qPCR technique, but that it cannot be taken for granted that inoculation with a well-colonizing and persistent *Pseudomonas* strain has a positive effect on yield of maize.

## Introduction

Plant growth-promoting rhizobacteria (PGPR) are able to facilitate plant nutrient acquisition and can also act as biocontrol agents by suppressing soil-borne diseases (Lucy et al., 2004). The mechanisms by which these bacteria act are multiple and diverse. PGPR can directly affect nutrient acquisition by transforming plant-unavailable forms of nutrients into plant-available forms e.g., by nitrogen (N_2_) fixation (Bashan and de-Bashan, 2010), solubilization of inorganic or mineralization of organic phosphorus (P) (Miller et al., 2010) or indirectly with promotion of root growth (Shaharoona et al., 2006; El Zemrany et al., 2007). Some PGPR are able to act as so called “mycorrhizal helper bacteria” and improve the colonization by arbuscular mycorrhizal fungi (AMF) which themselves are generally beneficial for plant nutrient status and health (Meyer and Linderman, 1986; Smith and Read, 2008). Besides affecting nutrient acquisition, production of antifungal substances and the competition for space and nutrients with pathogenic microorganisms are other ways to promote plant growth (Martinez-Viveros et al., 2010). PGPR can be formulated as microbial inoculants and their application in the field represents an interesting alternative to conventional applications of chemical fertilizers which can get lost in agricultural systems either by leaching (e.g., nitrogen) (Vitousek et al., 1997; Di and Cameron, 2002) or by absorption to metal complexes in the soil (e.g., phosphates) (Gyaneshwar et al., 2002). The application of PGPRs with P-solubilizing ability is compatible with low-input farming practices, which aim at reducing the use of these chemical fertilizers and promoting alternative sources of nutrients (e.g., rock-phosphate, organic fertilizers). Maize (*Zea mays*), the 3rd most cultivated cereal in the world (FAOSTAT, 2012), is particularly sensitive to low phosphorus availability (Postma and Lynch, 2011) and has shown responsiveness to PGPR applications (Gholami et al., 2009; Rosas et al., 2009; Walker et al., 2011). It is therefore an interesting crop to assess the potential of PGPR inoculants to improve phosphorus use efficiency and in this way to reduce the use of conventional P fertilizers. Nevertheless, the major constraint that prevents the large-scale application of PGPR in maize production is the inconsistency of their effects, which vary depending upon the inoculated bacterial strain, the formulation of the inoculant, the plant variety and the characteristics of the soil (Fuchs et al., 2000; Shaharoona et al., 2006; Egamberdiyeva, 2007). Nevertheless, the improved plant biomass and increased phosphorus acquisition by a plant after inoculation of a competent strain were found to be higher in soils which have a low microbial biomass and activity (Fliessbach et al., 2009; Mäder et al., 2011) and low levels of plant available phosphorus (Egamberdiyeva, 2007). Another reason explaining the inconsistency of these effects is explained by the poor survival of the inoculant in the soil (Khan et al., 2007). To establish, the introduced bacteria have to survive in the soil environment and be able to acquire sufficient quantities of soil and plant nutrients while competing with the indigenous soil community. Survival ability of PGPR is influenced by the abundance and the composition of the indigenous microbiota (Strigul and Kravchenko, 2006) but also by soil chemical and physical properties, such as pH, clay and soil organic matter content (Hartel et al., 1994; Bashan et al., 1995). In this context, the objective of the presented study was to develop a quantitative qPCR assay allowing the specific assessment of the colonization rate by two PGPR inoculants and enabling multiplex reactions. Furthermore, this tool was used to determine the influence of several factors on the survival rate of these PGPR when inoculated to maize in soils with varying pH, available P and microbial biomass. A secondary objective was to study the plant responses to PGPR application.

For these inoculation tests, we chose two strains of the genus *Pseudomonas*, an important genus of phosphate solubilizing bacteria (Rodríguez and Fraga, 1999). The strains studied were *Pseudomonas fluorescens* Pf153 (Fuchs et al., 2000) and *Pseudomonas* sp. DSMZ 13134 (Buddrus-Schiemann et al., 2010).

Our expectations were that better plant responses would be associated with higher levels of colonization by the inoculated PGPR strains and that the survival of these strains would be positively correlated with low soil microbial biomass, in particular with low abundance of soil native *Pseudomonas* communities. Finally, we tested the hypothesis that the improvement of plant nutrition and biomass due to inoculation would be more pronounced in soils with a low level of plant available nutrients and in particular with low level of plant available phosphorus.

## Materials and methods

### Tracing tools design

For the design of specific tracing tools, the following approach was used. First, the diversity of indigenous *Pseudomonas* rhizoplane isolates was determined by Matrix-Assisted Laser Desorption Ionization (MALDI) Time of Flight (TOF) mass spectrometry (MS) (Mulet et al., 2012). Then, representative isolates were sequenced and primer/probes specific for DSMZ 13134 were designed and cross-tested while an existing qPCR assay for Pf153 was further elaborated. This resulted in two qPCR assays enabling the specific tracing of the two strains in root and rhizosphere extracts of inoculated maize plants in multiplex assays. The details are given below.

#### Isolation of native Pseudomonas spp. from the maize rhizoplane and their characterization by MALDI-TOF MS

**Figure 1:**
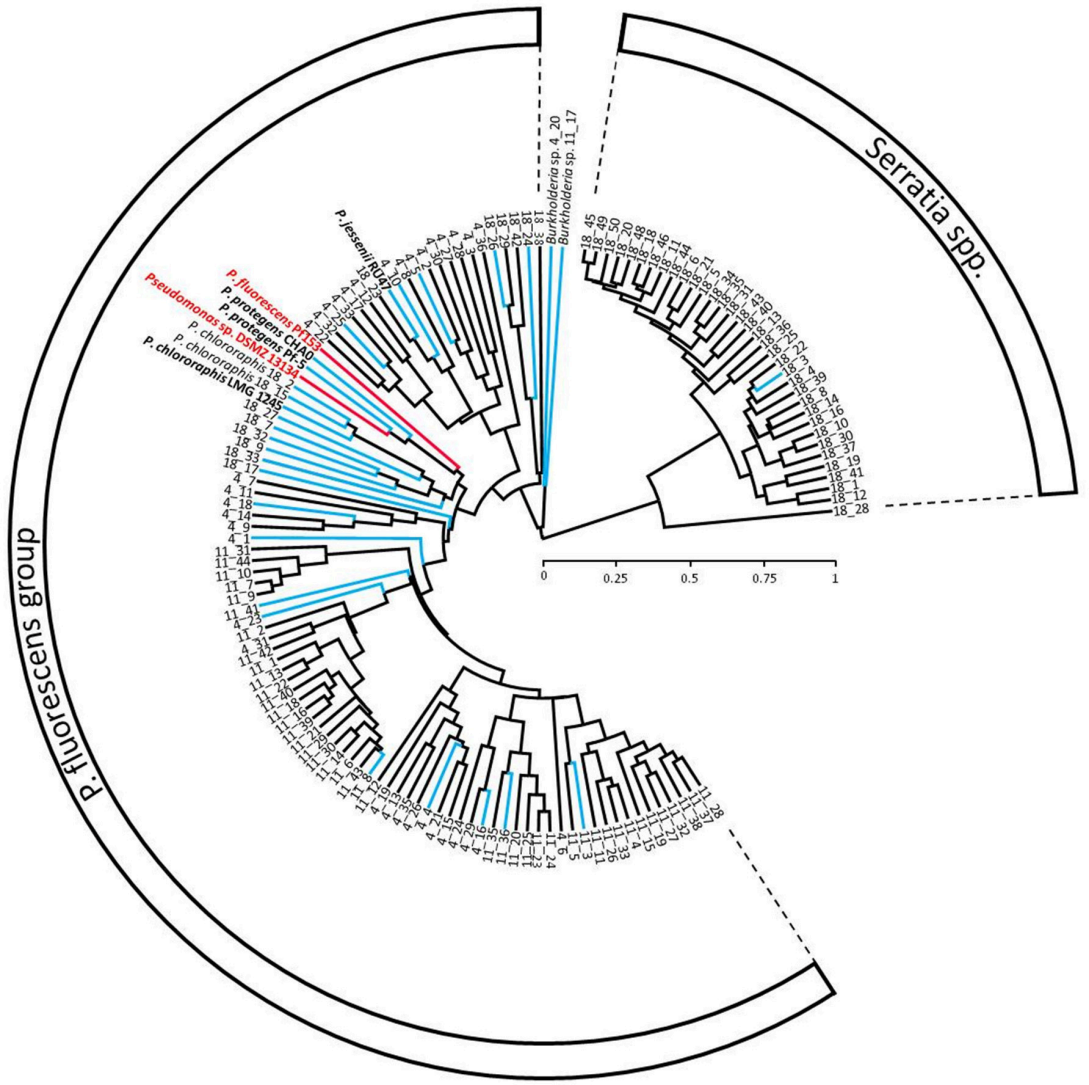
**MALDI-TOF cluster diagram with rhizoplane isolates from maize grown in soils DOK-M (4_1 to 4_36), Buus (18_1 to 18_50), and Le Caron (11_1 to 11_44) together with the target strains ***Pseudomonas fluorescens*** Pf153 and ***Pseudomonas*** sp. DSMZ 13134 (both in red) as well as ***Pseudomonas*** strains: ***P. jessenii*** RU47, ***P. protegens*** CHA0, ***P. protegens*** Pf-5 and ***P. chlororaphis*** LMG 1245**. Strains in blue were chosen for the cross-reactivity assay with the qPCR tracing tools developed for the target strains (in red). Identification of rhizoplane isolates is based on the SARAMIS™ database (Mabritec AG, Riehen). Scale: relative similarity of total mass number.

*Pseudomonas* spp. living in the rhizoplane of 4-week-old maize plants grown in the three soils were isolated. To access the rhizoplane, maize roots at harvest were washed with water at low pressure until no remaining soil adhered. Two grams of root material were then shaken in 50 ml sterile 0.9% NaCl at 150 rpm for 90 min. This suspension was filtered through glass wool (Typ N, Carl Roth, Germany) and the filtrate centrifuged at 3000 g for 15 min. The pellet was finally re-suspended in 1 ml sterile 0.9% NaCl (“rhizoplane wash”). Afterwards, serial dilutions of the rhizoplane wash were made with sterile 0.9% NaCl, and were plated on *Pseudomonas* selective King's medium B (KB) agar containing 40 μg per ml ampicillin, 13 μg per ml chloramphenicol and 100 μg per ml cycloheximide (Meyer et al., 2011) and incubated for 2 d at 25°C. Every colony of an appropriate dilution (around 40 per soil) were individually transferred into 500 μl liquid KB (King et al., 1954) in which *Pseudomonas* spp. were routinely grown and incubated for 1 d at 25°C. One 450 μl aliquot of each of these liquid cultures was mixed with 87% glycerol at a 1:1 (v/v) ratio and stored at −80°C, while the remaining 50 μl were used for species identification using MALDI-TOF MS and further analyzed and clustered with the SARAMIS™ database (Sauer and Kliem, 2010; Mulet et al., 2012) at Mabritec AG (Switzerland). Based on the cluster diagram (Figure 1), representative strains (25 in total) were chosen as controls in the cross-reactivity tests (see below) of the developed tracing tools. The first selection criterion for the choice of the 25 strains was to obtain a representative strain for each MALDI-TOF cluster, and secondly to select strains closely related to either *P. fluorescens* Pf153 or *Pseudomonas* sp. DSMZ 13134 that were used in the inoculation and tracing experiments.

#### Bacterial strains

All bacterial strains used in this study are listed in Table 1. Both target strains are efficient root colonizer (Buddrus-Schiemann et al., 2010; Von Felten et al., 2010) and are able to solubilize inorganic phosphate (Miller et al., 2010; Fröhlich et al., 2012). Strain *Pseudomonas sp*. DSMZ 13134 is the active component of the commercially available inoculation product Proradix® (Sourcon Padena, Germany). Strain Pf153 has been shown to produce antifungal compounds, such as cyanide and to act as biocontrol agent (Fuchs et al., 2000). Like strain Pf153 (Von Felten et al., 2010), strain DSMZ 13134 has been studied in maize and its application has been shown to improve plant biomass and P content (Nkebiwe et al., 2016) as well as in a number of other plant species (Yusran et al., 2009; Fröhlich et al., 2012).

**Table 1:** **Bacterial strains and isolates used in this study**.

**Genus**	**Species**	**Strain^a^**	**Origin**	**References**
**TARGET STRAINS**				
*Pseudomonas*	*fluorescens*	Pf153	Morens, Switzerland	Fuchs, 1993
*Pseudomonas*	sp.	DSMZ 13134	Hohenheim, Germany	Buddrus-Schiemann et al., 2010
**REFERENCE STRAINS**				
*Pseudomonas*	*jesseni*	RU47	United Kingdom	Adesina et al., 2007
*Pseudomonas*	*protegens*	CHA0	Morens, Switzerland	Stutz et al., 1986; Ramette et al., 2011
*Pseudomonas*	*protegens*	Pf-5	Texas, USA	Howell and Stipanovic, 1980; Ramette et al., 2011
*Pseudomonas*	*chlororaphis*	LMG 1245	Maas river, The Netherlands	LMG collection
**Genus**	**Species**	**Soil isolates^b^**	**Origin**	**References**
*Pseudomonas*	sp.	4_1, 4_4, 4_5, 4_10, 4_16, 4_18, 4_23, 4_25	Therwil (DOK-M), Switzerland	This study
*Burkholderia*	sp.	4_20	Therwil (DOK-M), Switzerland	This study
*Pseudomonas*	sp.	11_3, 11_8, 11_36, 11_41	Epiquerez (Le Caron), Switzerland	This study
*Burkholderia*	sp.	11_17	Epiquerez (Le Caron), Switzerland	This study
*Pseudomonas*	*chlororaphis*	18_2, 18_15	Buus, Switzerland	This study
*Pseudomonas*	sp.	18_7, 18_9, 18_17, 18_24, 18_26, 18_27, 18_32, 18_33	Buus, Switzerland	This study
*Serratia*	sp.	18_3	Buus, Switzerland	This study

a LMG, LMG bacteria collection, Gent, Belgium

b *Isolates in this study were identified by MALDI-TOF*.

#### Tracing tool development

Specific primers and a hydrolysis probe for *P. fluorescens* Pf153 (Table 2) were newly designed based on a previously described sequence-characterized amplified region (SCAR) (Gobbin et al., 2007) using the Beacon Designer software, v. 7.2. For tracing of *Pseudomonas* sp. DSMZ 13134, the *dnaX* gene was targeted analogous to Sławiak et al. (2009). To achieve this, DNA extracts of the target strain DSMZ 13134 and of 7 randomly chosen *Pseudomonas* soil isolates from DOK-M and Le Caron, as well as of the strains Pf153 and *P. jessenii* RU47, were obtained from pure KB cultures using the ZR Fungal Bacterial DNA Mini Prep™ kit (Zymo Research) following manufacturer's recommendations and using the FastPrep®-24 (MP Biomedicals) high-speed cell disruptor at a speed of 6 ms^−1^ for 40 s. These DNA extracts were used as templates for amplification of a 944 bp part of the *dnaX* gene using primers designed specifically for *Pseudomonas dnaX* with the online primer blast tool (http://www.ncbi.nlm.nih.gov/tools/primer-blast/) based on the alignment of *dnaX* sequences of different *Pseudomonas* strains available in the GenBank database (www.ncbi.nlm.nih.gov/genbank) and in the *Pseudomonas* Genome Database (www.pseudomonas.com). See Supplementary Material 1, Table 1 for primer sequences and PCR conditions. The PCR products were then purified using QIAquick PCR Purification Kit (Qiagen) and Sanger-sequenced by Microsynth AG (Switzerland). The obtained *dnaX* sequences (accession numbers: KP247587 to KP247596) were aligned together with sequences from the different databases with the Mega software package (v. 5.1) (Tamura et al., 2007) in order to identify unique and specific regions for the strain DSMZ 13134. Homologous *dnaX* sequences of non-target *Pseudomonas* strains were included to prevent selection of regions that would potentially lead to cross-reactions in the qPCR assay. Based on this alignment, primers and a hydrolysis probe (Table 2) were designed using Allele ID 6.0 (Premier Biosoft, USA). However, sequence comparison of the *dnaX* gene of DSMZ 13134 revealed complete congruence with *Pseudomonas protegens* type strain CHA0 and Pf-5 and close relatedness with *P. chlororaphis* strain LMG 1245. None of the native strains (isolated from the Buus, DOK-M and Le Caron soils) were found to be closely related to these reference strains [originating from Western part of Switzerland (CHA0) and the USA (Pf-5)] (Figure 1), indicating that the occurrence of these two reference strains was likely to be negligible in our experimental soils.

**Table 2:** **Primer and probe sequences of target strains and internal standard**.

**Primers and probes**	**Sequence (5′ → 3′)**	**Amplicon length (base pairs)**
Pf153_F	CGACCATCTTCAAGCCCTTGG	127
Pf153_R	GACGTTGGGACGGGTATTTCG	
Pf153_P	FAM-CCTCATGGCTACGTGGACCAATCACCTT-BHQ1^a^	
DSMZ13134_F	CTCTCCAGCCACTCGTTCAAC	174
DSMZ13134_R	GCTCAAGTGCTCCACCACC	
DSMZ13134_P	FAM-CGAAGAGCCGCCGCCCTACGTCAA-BHQ1^a^	
ACMV_F	CCACAGACAAGATCCACTCTCC	86
ACMV_R	CACTCTACTCAGGTTCCAATCAAAG	
ACMV_P	ROX-ACAGACAATTCAAGAAGCGAGCCATCCG-BHQ2^a^	

a *BHQ, Black Hole quencher*.

#### Calibration of the assays

**Figure 2:**
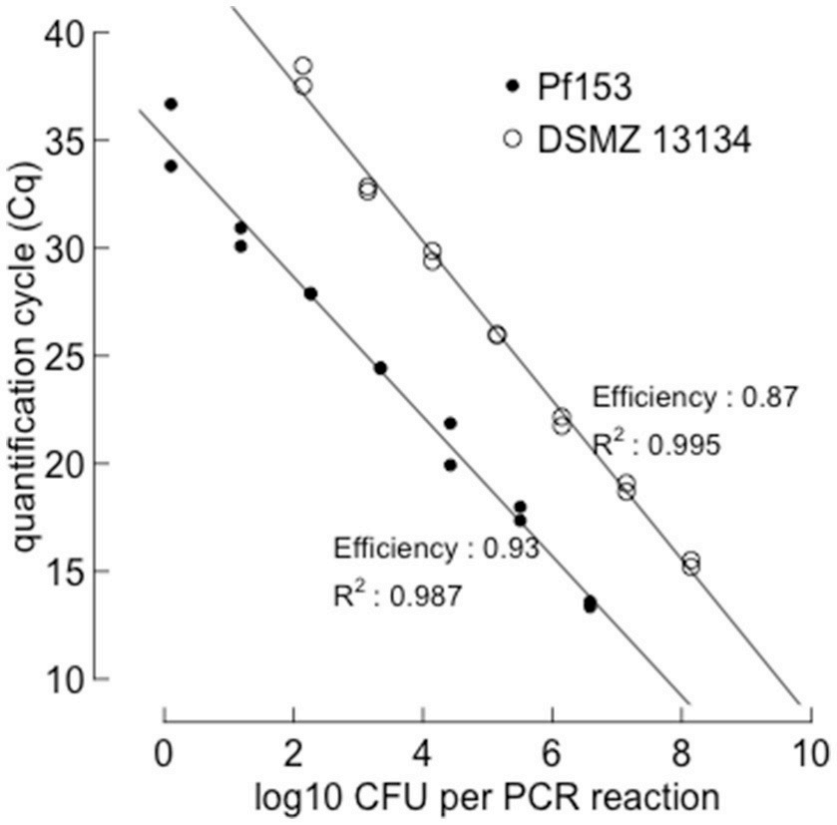
**qPCR standard curve for the tracing of ***Pseudomonas fluorescens*** Pf153 and ***Pseudomonas*** sp. DSMZ 13134**. Known amounts of copies of the target sequences were measured by quantitative PCR and calibrated in a second assay to colony forming units (CFU). An efficiency of 1.00 indicates that the amount of product doubles with every cycle.

To assess the number of gene copies per cell, target sequences for strains Pf153 and DSMZ 13134 were amplified (see Supplementary Material 1, Table 1 for primer sequences and PCR conditions) and the PCR products then purified (Qiaquick, Qiagen), blunted and ligated into a pJET1.2/blunt cloning vector, using the CloneJET PCR cloning kit (Thermo Scientific) following the manufacturer's recommendations. Then competent *E.coli* cells (Subcloning Efficiency™ DH5α™ competent *E. coli*, Invitrogen) were transformed and the plasmid DNA extracted using peqGOLD Plasmid Miniprep Kit II (Peqlab) according to the manufacturer's protocol. Copy numbers were calculated according to Thonar et al. (2012) after determination of the DNA concentration of the plasmid extracts and by knowing the size of the corresponding plasmid including the insert (3918 bp for DSMZ 13134 and 3121 bp for Pf153). Plasmid DNA solutions were serially diluted with TE buffer (1 mM Tris, 0.01 mM Na_2_EDTA, pH 8) to determine the detection level and were routinely used to calibrate the qPCR assays (see Figure 2). In a second step, a conversion factor that translated gene copy number into CFU was calculated. For this purpose, aliquots of 50 μl serially diluted (10^−5^ to 10^−8^) bacterial cultures were plated on KB agar. DNA from these serial dilutions was also extracted and measured with the qPCR markers using the different dilutions of the plasmids as calibration standards. CFU were counted after incubating the plates for 2 days at room temperature. Conversion values for translating target copy number to CFU were 57 and 6 for the targeted SCAR (*P. fluorescens* Pf153) and the *dnaX* fragment (*Pseudomonas* sp. DSMZ 13134), respectively.

#### Cross-reactivity and sensitivity testing

Cross-reactivity was tested with DNA extracts of pure cultures of reference *Pseudomonas* strains, isolated bacterial strains from the three soils (Table 1 and Figure 1) and with DNA extracts of root and rhizoplane of maize plants grown in the three experimental soils but also in seven additional soils. These seven additional soil materials derived from the DOK field trial (DOK-D2, DOK-K2 (see Mäder et al., 2000) for details of the DOK trial and its treatments)), from other arable fields in Switzerland (Buus ORG, Ettingen, Embrach, Full) and from a French site (Alsace). DNA extraction of pure cultures was performed by boiling 100 μl bacterial culture mixed with 900 μl lysis buffer (10 mM Tris, 1 mM EDTA, 0.5% Tween 20) according to (Kawasaki, 1990) (modified; without addition of Proteinase K) at 100°C for 10 min. The clear supernatant was used as template. Sensitivity and cross-reactivity of pure cultures was tested in simplex qPCR assays for strains DSMZ 13134 and Pf153 using 100 ng DNA per reaction as template and including a SYBR Green qPCR assay targeting the 16S rDNA region as a positive control (see Supplementary Material 1, Table 2 for sequences and assay conditions). DNA extractions as well as qPCR assays with root and rhizoplane templates were performed as described below.

Sensitivity of the markers was evaluated by assessing the detection limit of the plasmids used for calibration in a qPCR reaction (see above) and also by spiking different amounts of bacterial cells of the two strains in a rhizoplane matrix. For the latter, 20 μl of serially diluted cultures (with known CFU contents of the strains DSMZ 13134 and Pf153) were given to 250 μl rhizoplane wash spiked with 5 μl of the internal standard (3 × 10^8^ copies per μl). This internal standard, thereafter called ACMV, consisted of a plasmid carrying a fragment of cassava mosaic virus and was added to account for possible occurrence of PCR inhibitors or DNA losses during extraction. The same amount (20 μl) of sterile saline was used as a negative control. The experiment was performed in three replicates and DNA was extracted as described below. Samples were measured in a multiplex assay with the internal standard ACMV. Additional details are included in the “MIQE checklist,” following a proposal by Bustin et al. (2009) (Supplementary Material 2).

#### Quantitative PCR conditions

qPCR was performed with Rotor-Gene Q, Model 5-Plex HRM (Qiagen). The qPCR reaction was performed in a total volume of 12 μl using KAPA Probe Fast qPCR Kit Master Mix 2x Universal (Axonlab, Switzerland), containing 0.4 μM of each primer, 0.1 μM of the hydrolysis probe and 1 μl of the DNA template (approximatively 50 ng DNA for root DNA extracts and 10 ng DNA for rhizoplane DNA extracts). The reaction conditions for all qPCR assays (including for the internal standard ACMV, see Table 2 for primer and probe sequences) were established as follows: Initial enzyme activation at 95°C for 3 min, then 40 cycles consisting of denaturation at 95°C for 3 s, and annealing/extension at 60°C for 20 s.

### Pot experiment

The pot experiment compared the persistence of the two *Pseudomonas* strains in different soils and assessed their effect on maize performance. For this, maize of the organic variety Colisee (KWS Saat, Germany) was inoculated with *P. fluorescens* Pf153, and *Pseudomonas* sp. DSMZ 13134 (Table 1) and grown in the soils DOK-M, Buus and Le Caron (Table 3) together with a non-inoculated control. Each treatment was replicated eight times. Half of the replicates were harvested after 4 weeks and the second half after 8 weeks. The effect of seed inoculations on maize biomass, height, and nutrient uptake (N and P), as well as their influence on root colonization by native AMF was assessed. The soils DOK-M, Buus and Le Caron were chosen due to their contrasting parameters, such as pH, phosphorus content and management. The DOK-M soil belongs to the 36-year-old “DOK” system comparison trial (Mäder et al., 2000, 2002). This field was conventionally managed with only the addition of mineral fertilizers. The Buus soil originates from a field that has been organically managed (IFOAM, 2006; Demeter International e.V., 2012) for the past 30 years, while the Le Caron soil is derived from a 5-year-long grass/clover ley that was converted to a conventionally managed, no-tillage field in 2011.

**Table 3:** **Geographic origin, texture, pH, organic carbon content, phosphorus content and microbial community of the soils used in this study**.

**Soil**	**Geographic origin**	**Texture**			**pH**	**organic carbon**	**Phosphorus**		**Microbial community analyses^a^**				
		**Clay (%)**	**Sand (%)**	**Silt (%)**	**(CaCl_2_)**	**(%)**	**Ammonium-acetate EDTA-extractable P (mg P kg^−1^)**	**CO_2_ saturated water extractable P (mg P kg^−1^)**	**microbial carbon (μg per g dry weight)**	**microbial nitrogen (μg per g dry weight)**	**18S rDNA (copies per g dry weight)**	**16S rDNA (copies per g dry weight)**	**Native *Pseudomonas* (CFU per g root fresh weight)**
DOK-M	Therwil (Switzerland)	16.7	2.7	80.6	5.7	1.3	24.7 (medium^b^)	1.1	77.6 (c)	13.9 (b)	6.0 × 10 ^9^(a)	4.2 × 10 ^10^(b)	1.7 × 10 ^5^(b)
Buus	Buus (Switzerland)	29.9	3.9	66.2	6.6	2.6	17.5 (poor^b^)	0.6	207.0 (a)	34.5 (a)	4.3 × 10 ^9^(b)	7.3 × 10 ^10^(a)	2.2 × 10 ^5^(a)
Le Caron	Epiquerez (Switzerland)	29.9	3.5	66.6	4.8	2.4	5.8 (poor^b^)	0.2	140.3 (b)	15.4 (b)	3.9 × 10 ^9^(b)	4.4 × 10 ^10^(b)	4.3 × 10 ^4^(b)

*Microbial carbon and nitrogen, total fungal 18S rDNA copies and bacterial 16S rDNA copies were determined in the bulk substrate after 4 weeks of plant growth. CFU of native Pseudomonas was determined by plate counting of the rhizoplane of maize grown in the respective soils*.

a *different letters stand for significant differences of the microbial community analyses between the soils, ANOVA and Tukey's HSD test p < 0.05*.

b *Interpretation of soil P levels for crop growth in Swiss soils (Flisch et al., 2009)*.

#### Soil analyses and potting substrate

Texture (following the KGA-3 method VDLUFA, 1991) and soil pH (in CaCl_2_ Schinner et al., 1993) of the three soils were determined (Table 3). Ammonium-acetate EDTA-extractable and CO_2_-extractable P were measured at the Labor für Boden- und Umweltanalytik lbu (Switzerland). Microbial-bound carbon (C_mic_) and nitrogen (N_mic_) were determined by using the chloroform fumigation extraction method of Vance et al. (1987). For quantification of bacterial and fungal gene abundance, DNA was extracted from 0.3 g soil using the FastDNA Spin Kit for soil (Ref 116560200, MP Biomedicals). Bacterial (16S rDNA) abundance was quantified with the primers 314F and 534R (Muyzer et al., 1993) and fungal (18S rDNA) abundance with the primers ITS3F and ITS4R (White et al., 1990) in SYBR Green qPCR assays. qPCR conditions are based on Philippot et al. (2011). For detailed qPCR reaction and cycling conditions see Supplementary Material 1, Table 3. CFU number of native *Pseudomonas* (per gram of root fresh weight) was counted on the KB plates used for their isolation (see above for details on the isolation procedure).

For preparation of potting substrate, the three field soils were mixed with quartz sand (0.6–1.2 mm) (Trafor AG, Switzerland) in the ratio 2:1 (w:w) and incubated at 15°C (± 2°C) for 4 weeks prior to potting to allow stabilization of soil conditions. Plastic pots (3 L, Rosentopf Soparco, Hortima AG, Switzerland) were filled with the equivalent of 2.5 kg of this substrate as dry matter (DM). Each pot received the following nutrients: 33.3 mg N per kg DM substrate [source: Calcinit (17% N), provider: Yara Liva, Germany]; 55.3 mg K per kg DM substrate [source: Patentkali (25% K), provider: Hauert Dünger AG, Switzerland] and 16.7 mg P per kg DM substrate [source: Granuphos 18 (8% P), provider: Landor AG, Switzerland, 11% of this partially acidulated rock phosphate is water soluble P_2_O_5_]. Plants were grown in a chamber with 14 h light (using Hg/Na lamps; 30,000 lux) at 22°C and 10 h dark at 19°C.

#### Inoculation

Maize seeds were directly sown into pots and 5 ml of liquid bacterial inoculum was added to each seed in the planting hole. Control plants were inoculated with tap water. The inoculum was prepared by first growing the strains for 24 h in KB liquid at 25°C and 150 rpm. Secondly, 50 μl of this KB culture was transferred to 50 ml M1 media (Fuchs et al., 2000) and shaken over night at room temperature. Optical density was measured at 600 nm (UV/Vis) and the concentration adjusted to 3 × 10^8^ CFU per ml inoculum using the conversion factor of 1 × 10^8^ CFU per OD. A theoretical amount of 1.5 × 10^9^ CFU was therefore given to each planting hole. Exact CFU concentration of the inoculum was subsequently checked by plating on KB agar plates and counting the colonies after 2 days at 25°C. As a result, strains Pf153 and DSMZ 13134 were inoculated at a concentration of 5.0 × 10^8^ CFU and 3.6 × 10^8^ CFU per planting hole, respectively.

#### Maize response: measurements

Beginning with the second week, height was measured weekly during the 8-week growing period. After 4 and 8 weeks shoot biomass as well as root and rhizoplane samples were harvested. Shoot nitrogen and phosphorus content, as well as root biomass and AMF colonization were determined only after 8 weeks of plant growth. Shoot nitrogen concentration was measured using the NCS analyzer Flash EA 1112 (Thermo Electron Corporation) at ETH Zürich (Plant Nutrition Group) while shoot phosphorus concentration was determined spectrophotometrically after HCl extraction (Skalar Analytical B. V., 1995). AMF root colonization was calculated after a modified protocol of Phillips and Hayman (1970) and Brundrett et al. (1984). One hundred intersects were evaluated for the presence or absence of AMF structures.

#### Determination of strain persistence after DNA extraction from rhizoplane and root

DNA was extracted from the rhizoplane wash (see section Isolation of Native *Pseudomonas* spp. from the Maize Rhizoplane and their Characterization by MALDI-TOF MS for rhizoplane isolation) and from the roots remaining after the removal of the rhizoplane fraction. Therefore, the root compartment (henceforth called root) comprises bacteria living inside the root or attaching very efficiently to the root surface whereas the rhizoplane compartment (henceforth called rhizoplane) covers bacteria living on the roots but do not attach tightly and are washed off when shaken in saline water. The persistence of the *Pseudomonas* strains in these two compartments was measured in order to determine what the preferred site of colonization (root or rhizoplane) was. This information might explain, to some extent, the magnitude of plant growth effects of the strains.

Before DNA extraction the rhizoplane wash and the root homogenate were spiked with a known amount of the internal standard ACMV, a fragment of cassava mosaic virus (GenBank accession number AJ427910) (Thonar et al., 2012). For primer sequences and amplification conditions on how this ACMV plasmid was produced see Supplementary Material 1, Table 1. DNA extraction from rhizoplane was performed after a modified protocol from Llop et al. (1999). For the first step, 750 μl extraction buffer (200 mM Tris HCl pH 7.5, 250 mM NaCl, 25 mM Na_2_EDTA, 0.5% sodium dodecyl sulfate, 2% polyvinylpyrrolidone), 250 μl rhizoplane wash and 5 μl ACMV (with initial concentration of 4 × 10^8^ copies per μl) were heated in a 1.5 ml reaction tube at 95°C for 15 min and the remaining steps were done according to Llop et al. (1999). This DNA extract was resuspended in 100 μl of 0.1 × TE and further diluted 10 times for the qPCR reactions. DNA extraction from root (after removal of the rhizoplane fraction) was performed by following a modified protocol of Green et al. (1999). For this, 0.5 g root portion was homogenized in extraction bags (“Universal” 12 × 15 cm, Bioreba, Switzerland) using the Homex 6 (Bioreba, Switzerland) and by adding 5 ml of CTAB extraction buffer composed of 100 mM Tris, pH 8, 1.4 M NaCl, 50 mM Na_2_EDTA, 2% (w/v) CTAB, 1% (w/v) polyvinylpyrrolidone K25, 0.2% 2-mercaptoethanol. A known amount (2 × 10^9^ copies) of the ACMV plasmid was added to 500 μl of this root homogenate before DNA could be extracted with the DNAeasy plant Mini Kit (Qiagen) and eluted in a volume of 100 μl. The persistence of the strains was determined as described in section Quantitative PCR Conditions (quantitative PCR conditions).

### Statistics

Residuals of the data were tested for normality using the Shapiro-Wilk test and the following transformations were applied accordingly: arcsin for root length colonization, log10 for total *Pseudomonas* CFU and (log10)^−1^ for CFU of inoculated *Pseudomonas* sp. DSMZ 13134 and *P. fluorescens* Pf153. Two-way analyses of variance (ANOVA) were conducted with bacterial strain, soil, and their interaction as factors. If the interaction was significant, interpretations were made by testing the effect of each strain separately. In case of significant model effects, pair-wise mean comparisons using Tukey's HSD *post-hoc* test between the soil treatments were conducted. Any difference mentioned was found to be significant at *p* < 0.05. Statistical analysis was performed with RStudio Inc. (Version 0.97.551, © 2009–2012).

### Nucleotide sequence accession numbers

A 827-bp-long *dnaX* fragment from *Pseudomonas fluorescens* Pf153, *Pseudomonas* sp. DSMZ 13134, *Pseudomonas jessenii* RU47 and from the rhizoplane isolates *Pseudomonas* sp. 4_12, 4_25, 4_35, 11_3, 11_8, 11_11, and 11_14 was submitted to GenBank under accession numbers KP247587 to KP247596.

## Results

### Tracing tool: testing of cross-reactivity and sensitivity

The newly developed qPCR Taqman assays, based on a SCAR fragment for strain Pf153 and the *dnaX* gene for strain DSMZ 13134, allow a quantitative tracing of the two strains in the root and rhizoplane of maize.

Complete specificity of the tracing tool for Pf153 was confirmed (Table 4). As expected from the MALDI-TOF cluster diagram (Figure 1) and the *in silico* sequence comparison of the *dnaX* gene, the tracing tool for strain DSMZ 13134 showed a strong cross-reactivity with *Pseudomonas protegens* CHA0, *Pseudomonas protegens* Pf-5 and *Pseudomonas chlororaphis* LMG 1245. More than 10,000 times weaker amplification was observed with the isolates 18_2, 18_15 and 18_26 from the Buus soil. Nevertheless, no amplification was found to occur in the environmental samples of non-inoculated plants grown in the three soils used for the experiment as well as in seven additional soils (Table 4). All samples showed specific amplification of the internal standard ACMV with comparable quantification cycle values, which indicated that the DNA losses and/or the actions of PCR inhibitors were similar for all the DNA templates.

**Table 4:** **Cross-reactivity of the qPCR TaqMan assays**.

		**Strain/isolate name or origin of environmental sample**	**Tracing tool for *Pseudomonas* sp. DSMZ 13134^a^**	**Tracing tool for *Pseudomonas fluorescens* Pf153^b^**
			**CFU per 100 ng DNA**	
Pure cultures	Target strains	*P. fluorescens* Pf153	bdl^c^	2.9 × 10^7^
		*Pseudomonas* sp. DSMZ 13134	8.4 × 10^7^	bdl
	Strains tested for cross-reactivity	*P. protegens* CHA0	6.7 × 10^7^	bdl
		*P. protegens* Pf-5	1.0 × 10^8^	bdl
		*P. chlororaphis* LMG 1245	9.5 × 10^6^	bdl
		*P. jessenii* RU47	bdl	bdl
		*Pseudomonas* sp. 4_1, 4_4, 4_5, 4_10, 4_16, 4_18, 4_23, 4_25	bdl	bdl
		*Burkholderia* sp. 4_20	bdl	bdl
		*Pseudomonas* sp. 11_3, 11_8, 11_17, 11_36, 11_41	bdl	bdl
		*Pseudomonas* sp. 18_7, 18_9, 18_17, 18_24, 18_26, 18_27, 18_32, 18_33	bdl	bdl
		*P. chororaphis* 18_2	6.2 × 10^3^	bdl
		*P. chororaphis* 18_15	4.4 × 10^3^	bdl
		*Pseudomonas* sp. 18_26	4.1 × 10^2^	bdl
			**CFU per gram root fresh weight**	
Environmental samples	Rhizoplane of non-inoculated maize	DOK-M, Buus, Le Caron	Bdl	bdl
		DOK-D2, DOK-K2, Buus ORG, Ettingen, Alsace, Embrach, Full	Bdl	bdl
	Root of non-inoculated maize	DOK-M, Buus, Le Caron	Bdl	bdl
	other	Maize tissue	Bdl	bdl
		water	Bdl	bdl

*Cross-reactivity of the qPCR TaqMan assays for tracing of Pseudomonas sp. DSMZ 13134 and Pseudomonas fluorescens Pf153 with 100 ng DNA from liquid cultures of reference strains and of different soil isolates as well as from environmental rhizoplane and root samples of 4-week-old maize plants. Results with pure cultures are from the same experiment whereas results of environmental samples derive from different experiments*.

a *primers (DSMZ13134_F/ DSMZ13134_R) and probe (DSMZ13134_P)*;

b *primers (Pf153_F/ Pf153_R) and probe (Pf153_P)*;

c *bdl means below detection limit*.

*CFU means colony forming unit*.

The sensitivity of the two TaqMan assays was measured with 10-fold dilution series of the plasmid constructs holding the respective target sequences, as well as with bacterial cells (DSMZ 13134 and Pf153) that were serially diluted and spiked into a non-inoculated rhizoplane matrix. The detection limit for target DNA contained in the plasmid constructs was as few as 4 copies and 23 copies per μl reaction for Pf153 and DSMZ 13134, respectively. When bacterial cells were spiked into a rhizoplane matrix, the detection limits were 1.4 × 10^2^ CFU and 1.3 × 10^4^ CFU per gram root fresh weight for strain Pf153 and strain DSMZ 13134, respectively.

### Presence and persistence of inoculated *Pseudomonas* strains in the root and rhizoplane

**Figure 3:**
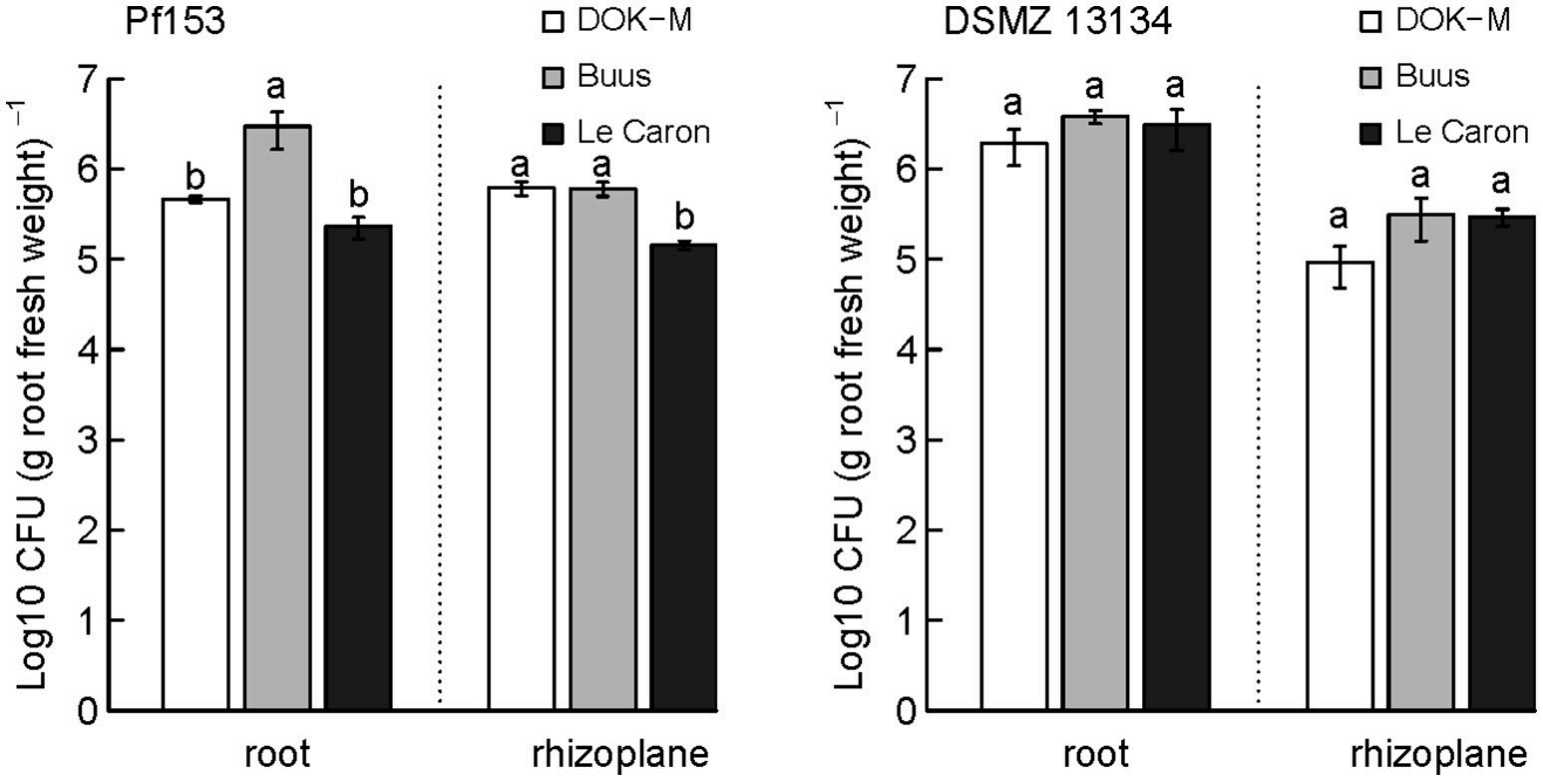
**Persistence of inoculated ***Pseudomonas fluorescens*** Pf153 and ***Pseudomonas*** sp. DSMZ 13134 in the root and the rhizoplane of 4-week-old maize plants in colony forming units (CFU) per gram root fresh weight**. ANOVA was calculated for rhizoplane and root separately. Tukey's HSD test, *p* < 0.05. *n* = 4, bars show mean values ± standard error.

The tracing tools for strains Pf153 and DSMZ 13134 were used to measure the bacteria's persistence in three contrasting soils and in two different compartments, the root and the rhizoplane (Figure 3).

Both strains were able to colonize the root and the rhizoplane of 4-week-old maize plants, but only strain Pf153 was still detectable after 8 weeks of growth (data not shown for persistence after 8 weeks). The persistence of strain Pf153 was influenced by the soil (*p* < 0.05; Figure 3) but not by the compartment or by the interaction between soil and compartment (*p* > 0.1 and *p* > 0.05, respectively). Population size of Pf153 in the root and the rhizoplane together was the highest for plants grown in the organic soil Buus (3.6 × 10^6^ CFU), followed by DOK-M (1.1 × 10^6^ CFU) and Le Caron (3.7 × 10^5^ CFU–in each case per gram root fresh weight). In contrast to strain Pf153, persistence of strain DSMZ 13134 was influenced by the compartment (*p* < 0.001) but neither by the soil and nor by the interaction of the two (*p* > 0.1). Colonization across all soils after 4 weeks was about 10 times higher in the root compared to the rhizoplane for strain DSMZ 13134.

After 8 weeks, population size of strain Pf153 was reduced about 3-fold for soils DOK-M and Le Caron and about 7-fold for soil Buus compared to week 4 (data not shown for week 8). As after 4 weeks, the persistence was higher for plants grown in soil Buus (4.8 × 10^5^ CFU), followed by DOK-M (3.0 × 10^5^ CFU) and Le Caron (1.2 × 10^5^ CFU–in each case per gram root fresh weight).

### Maize response

**Figure 4:**
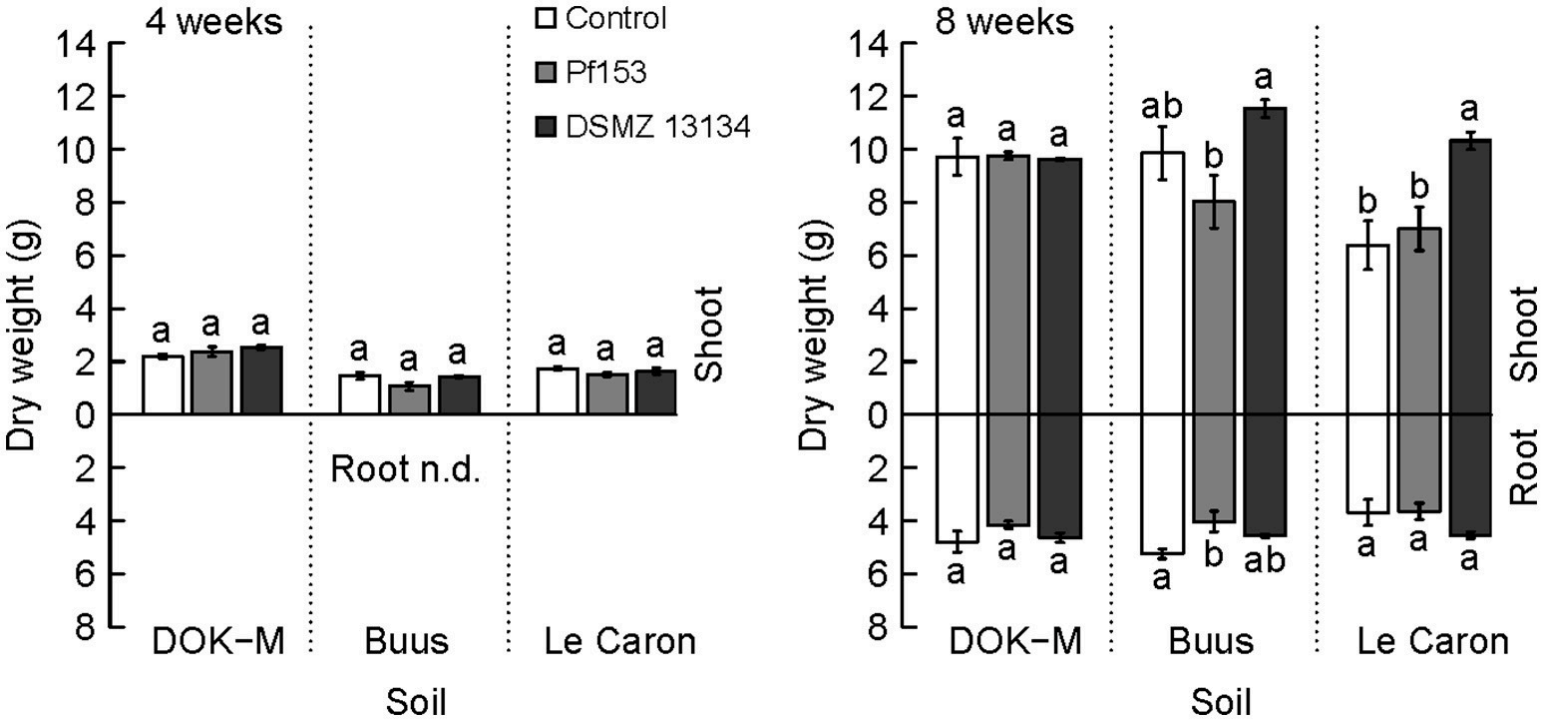
**Biomass of maize inoculated with ***Pseudomonas fluorescens*** Pf153 and ***Pseudomonas*** sp. DSMZ 13134. Left:** Shoot dry weight of maize after 4 weeks. Root dry weight was not determined (n.d.). **Right:** Shoot (above line) and root (below line) dry weight after 8 weeks of plant growth. ANOVA letters are calculated over each soil and for shoot and root separately. Tukey's HSD test, *p* < 0.05. *n* = 4, bars show mean values ± standard error.

**Figure 5:**
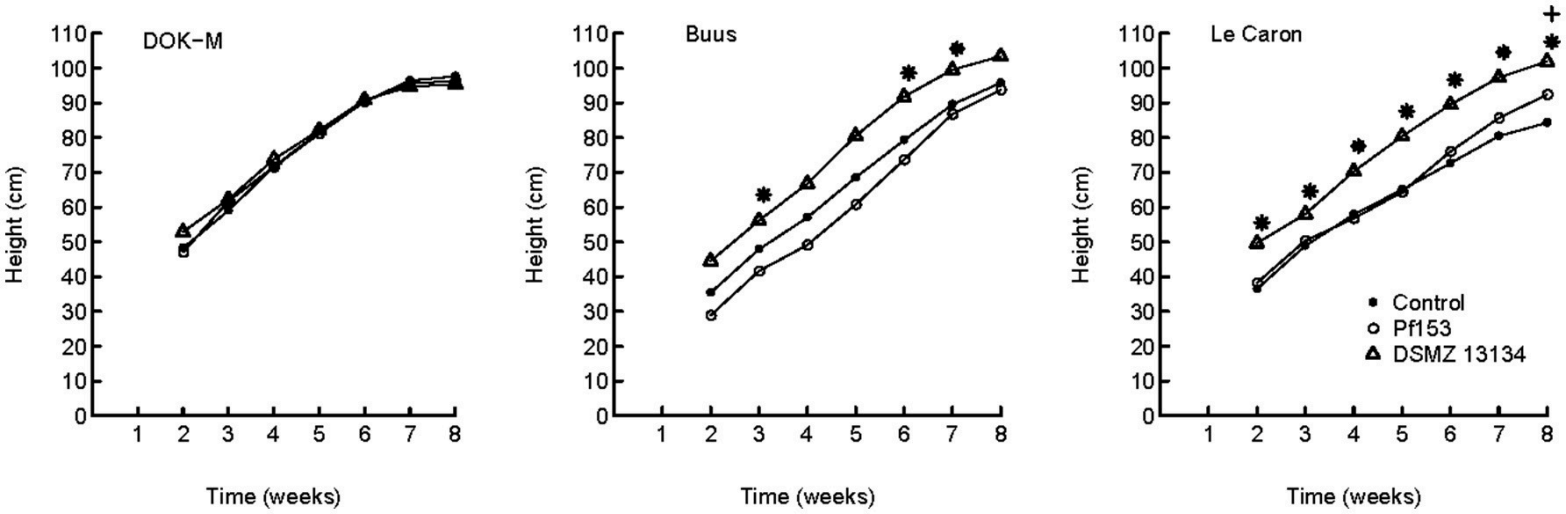
**Average height (cm) of maize plants grown in soils DOK-M, Buus and Le Caron from 2 to 8 weeks of plants harvested after 8 weeks (***n*** = 4)**. Plants were non-inoculated (Control) or inoculated with *Pseudomonas fluorescens* Pf153 or *Pseudomonas* sp. DSMZ 13134. Symbol ^*^ indicates time points with significant difference between strain *Pseudomonas* sp. DSMZ 13134 and the non-inoculated control treatment and symbol + between *Pseudomonas fluorescens* Pf153 and the non-inoculated control treatment. Pair-wise mean comparison (Tukey's HSD test, *p* < 0.05) was performed at each time point.

The response of maize upon the successful colonization up to week 4 (strain DSMZ 13134) and week 8 (strain Pf153) was analyzed. Strain Pf153 did not influence positively shoot biomass at any time or in any of the soils compared to non-inoculated plants (Figure 4). On the contrary, a 23% reduction of root biomass in the soil Buus compared to the non-inoculated plants was measured (*p* < 0.05) and a significant decrease in shoot biomass compared to plants inoculated with DSMZ 13134. Interestingly, strain DSMZ 13134 increased shoot biomass in the acidic and P-poor soil Le Caron by 62% compared to non-inoculated plants (*p* < 0.01; Figure 4) after 8 weeks. In this treatment, plant height was significantly increased already after 2 weeks of plant growth with, at week 8, a 21% increase in height compared to the non-inoculated plants (Figure 5). A stimulating growth effect on height, but not on biomass, was measured after 2, 6, and 7 weeks in soil Buus (*p* < 0.01).

None of the strains improved shoot nitrogen or phosphorus concentration of plants grown in any of the three soils (Supplementary Material 1, Figure 1). Nevertheless, the shoot P content of plants inoculated with DSMZ 13134 in the Buus soil was higher than in the other treatments. Shoot P and N concentrations of plants grown in Le Caron and inoculated with DSMZ 13134, where plant biomass was increased, were significantly reduced (Figure 4 and Supplementary Material 1, Figure 1).

Colonization by native arbuscular mycorrhizal fungi was not influenced neither by strain Pf153, known for its production of antifungal compounds (Fuchs, 1993; Fuchs et al., 2000) nor by strain DSMZ 13134, a known mycorrhizal helper bacterium (Yusran et al., 2009) in any of the soils (*p* > 0.1; Table 5). On average, plants grown in the Buus soil showed distinctly higher colonization by arbuscular mycorrhizal fungi (70%) compared to plants grown in either DOK-M (44%) or Le Caron soil (49%) (*p* < 0.001).

**Table 5:** **Root length colonization (%) by arbuscular mycorrhizal fungi of 8-week-old maize non-inoculated (Control) or inoculated with the two ***Pseudomonas*** strains when grown in the three soils**.

	**Across treatments**		**Control**		***Pseudomonas*** **sp. DSMZ 13134**		***Pseudomonas fluorescens*** **Pf153**	
DOK-M	44 ± 4	B^a^	42 ± 7	a^b^	46 ± 7	a	44±6	a
Buus	70 ± 4	A	69 ± 8	a	65 ± 5	a	75±10	a
Le Caron	49 ± 3	B	53 ± 6	a	48 ± 7	a	47±3	a

a *ANOVA was calculated across treatments (compare big letters vertically). Tukey's HSD test, p > 0.05. n = 12. Mean ± standard error shown*.

b *ANOVA was calculated for each soil separately (compare small letters horizontally). Tukey's HSD test, p < 0.05. n = 4. Mean ± standard error shown*.

## Discussion

### Detection limit and cross-reactivity of the qPCR assays

The qPCR tools developed for this study have been successfully applied for quantitative tracing of the two bacterial strains in environmental samples. The pre-characterization of native *Pseudomonas* related species with a MALDI-TOF MS approach has been instrumental to determine against which strains our tracing tools had to be cross-checked for absence of amplification. It has also helped to determine which native and reference strains should be included in the alignment of the target gene sequence for the design of strain specific markers. Von Felten et al. (2010) who established a SYBR Green qPCR assay on the same SCAR fragment in order to quantify Pf153 in the rhizosphere of maize, found a detection limit of 4 × 10^3^ CFU per g rhizosphere. While the detection limit for that same strain with our method is only slightly improved (1.4 × 10^2^ CFU per g root fresh weight), it allows a faster sample throughput with no need to assess dissociation temperature curves of the PCR products and it can be used in multiplex assays. The newly developed tracing tool for strain DSMZ 13134 is less sensitive compared to the tool developed for Pf153, having a 100 times higher detection limit. Still, the detection limit is comparable with other studies using qPCR tracing tools for environmental root and soil samples (Ibekwe and Grieve, 2003; Höppener-Ogawa et al., 2007; Savazzini et al., 2008). However, since the tracing tool for *Pseudomonas* sp. DSMZ 13134 is not entirely strain-specific, it is essential to include non-inoculated control samples in any assay it is used. Whether there is a high or negligible probability of cross-reacting with indigenous *P. protegens* or *P. chlororaphis* type bacteria present in soils, remains to be elucidated although in our soils such bacteria were not present or found in less than 2% of the cases for the taxa *Pseudomonas chlororaphis*.

### Persistence of inoculated *Pseudomonas* strains

Introduced bio-inoculants need to colonize the roots in sufficient numbers in order to express their plant growth promoting potential (Bull et al., 1991; Chin-A-Woeng et al., 2000). The data provided by the newly developed tools give further insight into the complex influences of physical, chemical and biological soil properties. In general, there was a trend toward better persistence of the inoculated strains in the Buus soil, which was statistically significant for the strain Pf153. The soil Buus was collected from an organically managed field site. Its crop rotation comprises 50% grass-clover, which increases soil organic carbon and microbial biomass. Therefore, the conditions for resource acquisition were better in that soil even though the data on the abundance of the indigenous microbiota (as assessed by the number of 16S copies per gram of soil and the population size of the native *Pseudomonas*, see Table 3) indicate a higher potential for competition with native populations. The high level of root colonization by AMF in this soil, which can be explained by its organic management (Brundrett, 1991; Jansa et al., 2002; Oehl et al., 2004), could have further facilitated the colonization by the strain Pf153 (Mayo et al., 1986; Meyer and Linderman, 1986; Barea et al., 1998). Studies on the influence of soil parameters on PGPR persistence are relatively scarce. Nevertheless, a positive correlation between the percentages of clay, total nitrogen, organic matter and water holding capacity and the survival of *Azospirillium brasilense* was found in a large screening of 23 soils (Bashan et al., 1995). In the present study, the latter three parameters (data for water holding capacity and total nitrogen not shown) were higher in the soil Buus, compared to DOK-M and Le Caron. The higher abundance of DSMZ 13134 in the root compared to the rhizoplane compartment shows that this strain attaches very efficiently or can even enter root crevices. This is in line with a study that demonstrated its preferred colonization site on the root surface of barley, and similarly to our results, colonization decreased after 3 weeks (Buddrus-Schiemann et al., 2010). On the other hand, Pf153 was also confirmed to be a good colonizer of maize (Von Felten et al., 2010). Its ability to produce cyanide could have conferred it a competitive advantage against the native microbiota (Fuchs, 1993; Compant et al., 2010). These results about persistence show that an active and rich soil like the Buus soil will not prevent root and rhizoplane colonization by PGPR. Nevertheless, more research is needed to define more precisely the set of soil conditions that enable a good persistence of introduced bio-inoculants.

### Growth response of maize after *Pseudomonas* inoculation

The rationale to inoculate plants with Pf153 and DSMZ 13134 is to improve biomass production and, to a lesser extent, nutrient acquisition also under P limiting conditions. Surprisingly, under our experimental conditions, strain Pf153 was not able to promote plant growth at all and in addition its increased colonization ability in soil Buus after 4 and 8 weeks was even associated with reduced plant biomass. The production of cyanide by Pf153, as reported in the literature (Fuchs et al., 2000), may have negatively affected plant growth directly, as it has been shown in other studies (Alstrom and Burns, 1989; Kremer and Souissi, 2001), as well as reduced the growth of indigenous beneficial microorganisms living in the maize rhizosphere and thereby negatively affected plant growth indirectly (Zdor, 2015). According to Kremer and Li (2003), the ability of cyanide producing rhizobacteria to affect other soil microorganisms is also related to the soil management with higher cyanide activity in soil receiving organic input and submitted to reduced tillage. This is the reason why many studies are now concentrating on side effects of PGPR inoculation, looking at their impact on the soil and rhizospheric microbial communities (Martinez-Viveros et al., 2010; Vacheron et al., 2013; Omirou et al., 2016; Schlaeppi et al., 2016). In contrast to Pf153, strain DSMZ 13134 was shown to significantly improve maize biomass in one of the studied soils. As hypothesized, this plant growth-promoting effect was observed in a soil with low soil P level (Le Caron). The other two soils had either a higher level of directly plant available phosphorus (DOK-M) or a higher level of phosphorus contained in the microbial pool (Buus). In these two soils, the inoculation could not further improve plant performance. The effectiveness of DSMZ 13134 in a nutrient-poor soil compared to a soil supplied with very high amounts of fertilizers was also found to be higher in a greenhouse experiment with barley (Fröhlich et al., 2012). In another study, strain DSMZ 13134 was able to increase tomato biomass and P content in a soil supplied with relatively high amounts of P (156 kg P ha^−1^) (Yusran et al., 2009). Nevertheless, the effectiveness of DSMZ 13134 in the Le Caron soil supports the trend that PGPR are more effective in unfertile, stressed soils or in soils with poor microbial biomass and activity (Strigul and Kravchenko, 2006; Fliessbach et al., 2009; Mäder et al., 2011). The mechanisms by which DSMZ 13134 improved maize biomass in the Le Caron soil remain to be elucidated. Indeed, in none of the three soils root colonization by AMF was improved even though DMSZ 13134 has been shown to be a mycorrhizal helper strain (Yusran et al., 2009). In other studies, that same strain has shown ability to dissolve insoluble tricalcium phosphate in *in vitro* conditions (Fröhlich et al., 2012). It is unlikely that this property is of any importance in the acidic soil of Le Caron (where phosphate is more bound to iron and aluminum Gyaneshwar et al., 1998, 2002; Jones, 1998). In Buus, a soil with a more alkaline pH and higher soil organic carbon and higher microbial P content compared to Le Caron, DSMZ 13134 improved the maize plant P acquisition. This might be attributed to P mineralization from the organic P pool (as shown in Fröhlich et al., 2012) and/or to mobilization of phosphate from insoluble mineral phosphate provided by the added rockphosphate. Our results indicate therefore that the outcome of bacterial inoculations is highly dependent on soil parameters. In our case, we saw a plant growth promoting effect of DSMZ 13134 in the Le Caron soil, which had a low pH and a low level of plant available phosphorus.

In conclusion, the beneficial effect strain DSMZ 13134 had on maize plants in the acidic and poor soil was not associated with a better survival or persistence, despite the fact that this persistence is likely a prerequisite for the realization of its plant growth promotion potential. However, it was shown that strain Pf153 had a negative effect on plant biomass in one of the three soils, although its improved ability to colonize the roots in that same soil. Hence, the importance of long term persistence of an introduced PGPR strain on its effects on plant growth might be overestimated. The tools developed in this study will be useful for future monitoring of the persistence and spread of these strains in pot and field experiments. Future efforts should be made to elucidate the required colonization levels as well as the traits and mechanisms that are important for rhizosphere colonization and plant growth promotion by specific PGPR in order to understand the conditions under which they can be successfully applied to the soil.

## Author contributions

CT, CM, and TO wrote the manuscript. CT, CM, and TO performed the molecular analyses. DZ performed the MALDI-TOF analysis. DN performed analytical analyses. CT, CM, TO, TB, EK, and PM contributed to the concept of this study.

## Funding

This study was funded by the BIOFECTOR project (Resource preservation by application of BIOefFECTORs in European crop production, grant agreement number 312117) under the 7th Framework Program (FP7), European Commission, Brussels, Belgium.

### Conflict of interest statement

The authors declare that the research was conducted in the absence of any commercial or financial relationships that could be construed as a potential conflict of interest.
