# Supplementary material for: Tracing of Two Pseudomonas Strains in the Root and Rhizoplane of Maize, as Related to Their Plant Growth-Promoting Effect in Contrasting Soils
Source: Front Microbiol. 2017 Jan 10;7:2150. doi: 10.3389/fmicb.2016.02150 (PMC5222796; doi:10.3389/fmicb.2016.02150)
Supplement: Supplementary file 1 [file Table1.docx]

***Supplementary Material 1***

**Tracing of two Pseudomonas strains in the root and rhizoplane of maize, as related to their plant growth-promoting effect in contrasting soils**

**Mosimann Carla ^1,2^, Oberhänsli Thomas ^2^, Ziegler Dominik ^3^, Nassal Dinah ^4^, Kandeler Ellen^4^, Boller Thomas ^1^, Mäder Paul ^2^, Thonar Cécile ^2*^**

*^1^ Zürich-Basel Plant Science Center, University of Basel, Department of Environmental Sciences, Botany, Basel, Switzerland
^2^ Research Institute of Organic Agriculture (FiBL), Frick, Switzerland*

*^3^ Mabritec AG, Riehen, Switzerland*

*^4^ University of Hohenheim, Institute of Soil Science and Land Evaluation, Stuttgart, Germany*

**^*^Correspondence:** Dr. Cécile Thonar**:** cecile.thonar@gmail.com, cecile.thonar@fibl.org

**1 Supplementary Tables**

**Supplementary Table 1**. Additional PCR protocols used in the study.

PCR protocol for amplification of the *dnaX* fragment of *Pseudomonas* sp., the sequence-characterized amplified region (SCAR) fragment and the ACMV fragment

| Primer sequences | | | | |
| --- | --- | --- | --- | --- |
| Target | Orientation | Sequence (5’→ 3’) | Amplicon length | Reference |
| *dnaX* fragment | forward | GGCCAGACSCATGTGCTCAA | 944 bp | This study |
|  | reverse | CCRATCAGGCCCATCTGRTA |  |  |
| SCAR fragment | forward | ACCTGCCTGCTCATTTTCCC | 137 bp | This study |
|  | reverse^*^ | GACGTTGGGACGGGTATTTCG |  |  |
| ACMV fragment | forward | GAAGGAGCAGGCTTTGGTTA | 945 bp | Krause HM (unpublished) |
|  | reverse | CGTCGATCCCCACTACCTAA |  |  |

^*^ Corresponds to Pf153_R

| PCR reaction mixture | |  | |
| --- | --- | --- | --- |
| PCR reaction buffer S 10x (Peqlab, Germany) | | 2.5 µl | |
| Primers | | 0.2 µM (each) | |
| dNTP Roti Mix PCR 3 (Carl Roth, Germany) | | 0.2 mM (each) | |
| Hot Taq DNA polymerase (Peqlab, Germany) | | 1 U | |
| Template (10 ng DNA of bacterial culture per µl) | | 2.5 µl | |
|  | |  | |
| Total reaction volume | | 25 µl | |
| PCR cycling conditions | | |  |
| Initial denaturation | 95°C | 15 min |  |
| Denaturation | 94°C | 30 s | 33 cycles |
| Annealing | *dnaX*: 56°C,  SCAR: 60°C  ACMV: 58°C | 30 s |  |
| Extension | 72°C | 1 min |  |
| Final extension | 72°C | 3 min |  |

Aliquots of 2 µl of the amplified PCR products were separated on a 1.5% (w/v) agarose gel containing RedSafe™ Nucleic Acid Staining (10^-4^ dilution, Intron Biotechnology) for visualisation of DNA bands under UV 366 nm.

**Supplementary Table 2.** PCR protocol for amplification of the 16S rDNA in a SYBR Green assay.

| Primer sequences for amplification of 16S rDNA^a^ | | | | |
| --- | --- | --- | --- | --- |
| Target | Primer name | Sequence (5’→ 3’) | Amplicon length |  |
| 16S rDNA | 341F | CCTACGGGAGGCAGCAG | 466 bp | |
|  | 797R | GGACTACCAGGGTATCTAATCCTGTT |  |  |

| qPCR reaction mixture for amplification of 16S rDNA^a^ | |
| --- | --- |
|  | Volumes/reaction (µl) |
| KAPA SYBR Fast qPCR Kit Master Mix 2x Universal (Axonlab) | 6 |
| Forward Primer (2 µM) | 0.5 |
| Reverse Primer (2 µM) | 1.4 |
| Water | 3.1 |
| Template (10 ng DNA of bacterial culture per µl) | 1 |
| Total reaction volume | 12 |

| qPCR cycling conditions for amplification of 16S rDNA^a^ | | | |
| --- | --- | --- | --- |
| Initial denaturation | 95°C | 3 min |  |
| Denaturation | 95°C | 10 s | 35 cycles |
| Annealing | 61.5°C | 20 s |  |
| Extension | 72°C | 20 s |  |
| Melting curve |  |  |  |
| Initial denaturation | 95°C | 1 min |  |
|  | 1° C increase every 10 s from 55°C to 95°C | |  |

^a^  after Muyzer et al. (1993) and Nadkarni et al. (2002)

As positive control and standard calibrator a plasmid carrying the 16S rDNA insert (466 bp) of *Pseudomonas jessenii* R62 (Mäder et al. 2011) in a pJET/blunt cloning vector (Thermo Scientific) was used (pers. comm. Krause HM, FiBL).

**Supplementary Table 3.** PCR protocol for amplification of the bacterial (16S) and fungal (18S) rDNA in a SYBR Green assay after Philippot et al. (2011).

| PCR reaction mixture for quantification of bacterial (16S rDNA) abundance with the primers 314F and 534R^a^ and fungal (18S rDNA) abundance with the primers ITS3F and ITS4R^b^ | |
| --- | --- |
|  | Volumes/reaction (µl) |
| DNA (5 ng per µl) | 1.5 |
| Forward primer (10 mM) | 0.75 |
| Reverse primer (10 mM) | 0.75 |
| Power SYBR Green PCR Mastermix (Applied Biosystems) | 7.5 |
| T4 gene 32 protein | 0.375 |
| Ultra-pure water | 4.125 |
| Total reaction volume | 15 |

| PCR cycling conditions for quantification of bacterial (16S rDNA) abundance with the primers 314F and 534R^a^ and fungal (18S rDNA) abundance with the primers ITS3F and ITS4R^b^ | | | |
| --- | --- | --- | --- |
|  | 16S rDNA | 18S rDNA |  |
| Initial denaturation | 95 °C – 10 min | 95 °C – 10 min |  |
| Denaturation | 95 °C – 15 s | 95 °C – 15 s | 35 cycles |
| Annealing | 60 °C – 30 s | 55 °C – 30 s |  |
|  | 72 °C – 30 s | 72 °C – 30 s |  |
| Extension | 75 °C – 30 s | 76 °C – 30 s |  |
| Melting curve | 95 °C – 15 s | 95 °C – 15 s |  |
|  | 60 °C – 1 min | 60 °C – 1 min |  |
|  | 95 °C – 15 s | 95 °C – 15 s |  |
|  | 60 °C – 15 s | 60 °C – 15 s |  |

^a^ after Muyzer et al. (1993)

^b^ after White et al. (1990)

**2 Supplementary Figure**


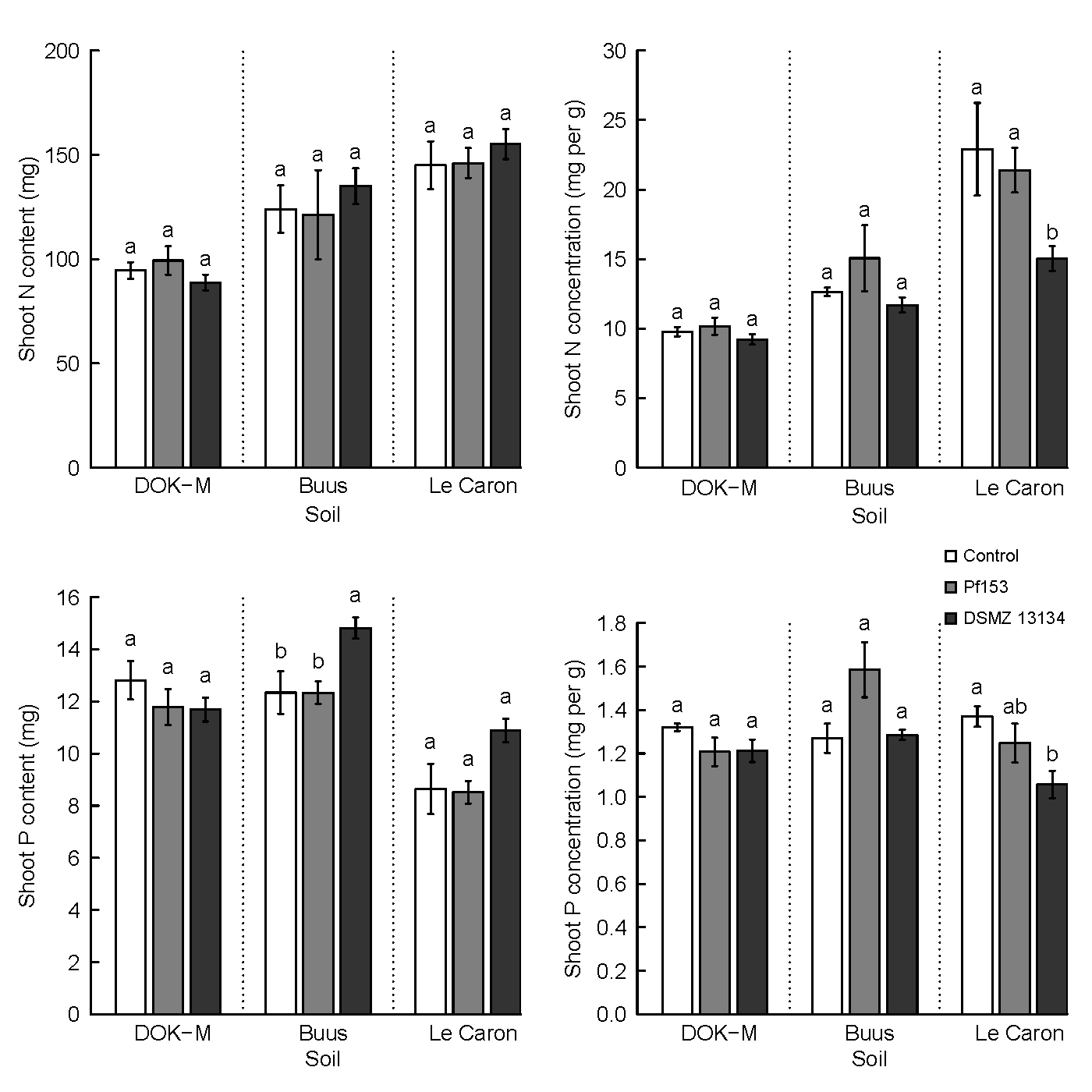


**Supplementary Figure 1.** Nitrogen and phosphorus content and concentration of 8-week-old maize grown in soils DOK-M, Buus and Le Caron.

Plants were non-inoculated (Control) or inoculated with *Pseudomonas fluorescens* Pf153 or *Pseudomonas* sp. DSMZ 13134. Nitrogen and phosphorus content in mg per plant (left) and concentration (right) in mg per g shoot dry weight. ANOVA letters are calculated over each soil separately. Tukey’s HSD test, p < 0.05. N = 4 (except N = 3 for treatments Pf153-inoculated Buus and control Le Caron), bars show mean values ± standard error.

**References**

Mäder, P., Kaiser, F., Adholeya, A., Singh, R., Uppal, H.S., Sharma, A.K., Srivastava, R., Sahai, V., Aragno, M., Wiemken, A., Johri, B.N., Fried, P.M. (2011) Inoculation of root microorganisms for sustainable wheat-rice and wheat-black gram rotations in India. *Soil Biol Biochem* 43:609-619. doi:10.1016/j.soilbio.2010.11.031

Muyzer, G., de Waal, E.C., Uitterlinden, A.G. (1993) Profiling of complex microbial populations by denaturing gradient gel electrophoresis analysis of polymerase chain reaction - amplified genes coding for 16S rRNA. *Appl Environ Microbiol* 59: 695-700.

Nadkarni, M.A., Martin, F.E., Jacques, N.A. and Hunter, N. (2002) Determination of bacterial load by real-time PCR using a broad - range (universal) probe and primers set. *Microbiology* 148: 257–266. doi: 10.1099/00221287-148-1-257

Philippot, L., Tscherko, D., Bru, D., Kandeler, E. (2011) Distribution of high bacterial taxa across the chronosequence of two alpine glacier forelands. *Environ Microbiol* 61, 303-312. doi: 10.1007/s00248-010-9754-y

White, T.J., Bruns, T., Lee, S. and Taylor, J. (1990) Amplification and direct sequencing of fungal ribosomal RNA genes for phylogenetics, in *PCR Protocols: A Guide to Methods and Applications* (M. Innis, D. Gelfand, J. Sninsky and T. White, eds.) 38, Academic Press. New York. 315-322. doi:10.1016/B978-0-12-372180-8.50042-1
